# Novel risk-factor analysis and risk-evaluation model of falls in patients receiving maintenance hemodialysis

**DOI:** 10.1080/0886022X.2023.2182608

**Published:** 2023-03-01

**Authors:** Xiaomin Liu, Sijie Chen, Caifei Liu, Xilong Dang, Meng Wei, Xia Xin, Julin Gao

**Affiliations:** a Department of Dialysis, The First Affiliated Hospital of Xi'an Jiaotong University, Xi'an, People’s Republic of China; b Medical School of Yan'an University, Xi'an, People’s Republic of China; c Nursing Department, The First Affiliated Hospital of Xi'an Jiaotong University, Xi'an, People’s Republic of China

**Keywords:** Fall, maintenance hemodialysis, depression, nomogram

## Abstract

This study investigated the prevalence of falls in maintenance hemodialysis (MHD) patients, and established a nomogram model for evaluating the fall risk of MHD patients. This study enrolled 303 MHD patients from the dialysis department of a tertiary hospital in July 2021. The general data of the participants, as well as the scores on the FRAIL scale, Sarcopenia Screening Questionnaire (SARC-F), Short Physical Performance Battery (SPPB) Scale, and of anxiety and depression, and the occurrence of falls were recorded. Using R language, data were assigned to the training set (*n* = 212) and test set (*n* = 91), and a logistic regression model was established. The regression model was verified by the receiver operating characteristic (ROC) curve, area under the curve (AUC), and the calibration curve. As a result, the prevalence of falls in MHD patients was 20.46%. Risk factors for falls in the optimal multivariate logistic regression model included hearing impairment, the depression score, and the SPPB score, of which a higher depression score (odds ratio (OR): 1.28, 95% confidence interval (CI): 1.09–1.49, *p* = 0.002) and SPPB ≤ 6 (OR*_vs_*_SPPB9-12_: 3.69, 95% CI: 1.04–13.14, *p* = 0.043) conferred independent risk for falls. AUC of the nomogram in the training was 0.773, which in the test group was 0.663. The calibration and standard curves were fitted closely, indicated that the evaluation ability of the model was good. Thus, a higher depression score and SPPB ≤ 6 are independent risk factors for falls in MHD patients, and the nomogram with good accuracy and discrimination that was established in this study has clinical application value.

## Introduction

As one of the first-line treatments for patients with kidney failure, 84% of kidney failure patients are currently receiving maintenance hemodialysis (MHD) treatment [1]. Long-term hemodialysis may cause many complications, such as kidney osteopathy, electrolyte imbalance, malnutrition, sarcopenia, and frailty [2–4]. As a result, the prevalence of falls in patients with chronic kidney disease (CKD) was 37.4%, even higher than that in the adults aged ≥65 years of 27.5% [5,6]. Falls related injury seriously decreases the quality of life of patients, increases the familial and societal burdens, and even causes death [7,8]. Identification of high-risk groups by using risk assessment tools can effectively decrease the risk of falls [9].

During the past few decades, many fall risk prediction tools, such as the Morse Fall Scale (MFS) and the Hendrich Fall Risk Assessment Model (HFRM-II), have been developed for the general older population. However, falls have a multifactorial causation including the physiological, pathological, psychological, and environmental factors. Specific assessment tools are needed to identify MHD patients who have a high risk for falls. Although many studies have discussed sarcopenia, weakness, or depression in MHD patients [10,11], few studies translated their findings into a clinically model to evaluate the risk of falls in MHD patients.

This study aimed to investigate the prevalence of falls in MHD patients, analyze the risk factors for falls, and build a risk evaluation model, to provide a basis for the development of targeted early-intervention measures for MHD patients.

## Methods

### Study design

This study was a cross-sectional study. A total of 303 patients with MHD in the dialysis department of the First Affiliated Hospital of Xi'an Jiaotong University in July 2021 were investigated. Whether the subjects had fallen in the past year was retrospectively collected through a questionnaire.

### Participants

The participants of this study were MHD patients who were treated in the dialysis department. The inclusion criteria were as follows: age ≥18 years; regular hemodialysis treatment ≥3 months; dialysis treatment 2–3 times/week; willingness to participate in this study, cooperating to complete the study questionnaire, and provision of informed consent for study participation. The exclusion criteria were as follows: presence of cognitive impairment or severe mental illness; blindness; stroke sequelae with limb motor dysfunction; severe cardiopulmonary complications; diagnosis of malignancy; limited self-care ability (Barthel index score ≤ 40) [12].

This study was approved by the hospital ethics committee (XJTU1AF2022LSK-127) and complies with the principles of the Declaration of Helsinki. All subjects signed informed consent.

### Data collection

Participant characteristics, including age, gender, education, duration of dialysis, height, weight, using of walking aids, hypertension, diabetes, hyperparathyroidism, intradialytic hypotension (IDH), visual impairment, hearing impairment, weakness, anxiety, depression, sarcopenia, and body function were recorded. The fall events of the participants in the past one year were ascertained.

The questionnaire of this study was completed by three uniformly trained investigators. Investigators conducted on-site questionnaire survey, completed and recovered on the spot. All questionnaires and physical function tests were completed 30 min before dialysis.

### Definition of key variables

Fall was defined as unintentional falls on the ground or other planes, but excluded physical falls due to loss of consciousness, violence, hemiplegia, or seizures [13].

IDH was defined according to the 2002 K/DOQI (Kidney Disease Outcomes Quality Initiative) as a decrease in systolic blood pressure ≥20 mmHg, or a decrease in mean arterial pressure ≥10 mmHg, with or without symptoms of hypotension, namely, abdominal discomfort, nausea and vomitus, syncope, etc. or any condition in need of medical intervention [14].

Anxiety and depression were assessed by the Hospital Anxiety and Depression Scale (HADS) [15].

Sarcopenia was screened using the Simple Five-item Scoring Questionnaire (SARC-F), which is recommended by the European Working Group on Sarcopenia in Older People [16] and the Asian Working Group for Sarcopenia [17], wherein a score of 0–4 and ≥5 represents a low and high risk of sarcopenia, respectively.

Body function was assessed by the Short Physical Performance Battery (SPPB), developed by the National Institute on Aging (NIA) [18] (Supplementary File 1). SPPB scores of 0–6, 7–9, and 10–12 indicated poor, moderate, and good body balance, respectively.

### Statistical analysis

Data analysis was performed using R (R for Windows Version 4.1.2, R Foundation for Statistical Computing, Vienna, Austria). Using ‘sample’ function in R, the data were divided into the training set (*n* = 212) and test set (*n* = 91) in a 7:3 distribution. Continuous variables were tested for normality using the Kolmogorov–Smirnov test. Normally distributed variables were expressed as the mean (SD). Abnormally distributed continuous variables were expressed as median [Q1–Q3], and intergroup comparisons were performed using the Mann–Whitney *U* tests. Categorical variables were expressed as the count (percentage), and comparisons between groups were performed using Pearson’s chi-square or Fisher’s exact test. Univariate logistic regression analyses were used to identify risk factors for falls. Factors with a *p* value less than 0.20 were included in the multivariable logistic regression. The optimal logistic model was selected according to the Akaike information criterion (AIC). AIC is a tool to evaluate the performance of models, which lower AIC means better model [19]. Receiver operating characteristic (ROC) curves as well as the area under the curve (AUC) and calibration curves were constructed. Power analysis was performed using package ‘pwr’. *p* < 0.05 indicated statistical significance (two-sided).

## Results

### Baseline characteristics

This study enrolled 303 participants, and the prevalence of fall in MHD patients was 20.46%. The baseline characteristics are presented in Table 1. Participants in the fall group were older, lower grip strength, higher rate of comorbidities, and poorer body function.

**Table 1.** Baseline characteristics of the participants.

Variable	No fall (*N* = 241)	Fall (*N* = 62)	*p* Value
Age, years (IQR)	51 [39;64]	62 [51.5;70]	<0.001
Gender (*N* (%))			0.183
Male	158 (65.56%)	35 (56.45%)	
Female	83 (34.44%)	27 (43.55%)	
Duration of dialysis, months (IQR)	48 [24;72]	48 [24;81]	0.423
Educational level (*N* (%))			0.016
Primary school	17 (7.05%)	6 (9.68%)	
Junior high school	43 (17.84%)	23 (37.1%)	
Senior high school	57 (23.65%)	10 (16.13%)	
College or higher	124 (51.45%)	23 (37.1%)	
BMI, kg/m^2^ (IQR)	22 [20;25]	22.5 [20;25]	0.928
DM (*N* (%))	63 (26.14%)	24 (38.71%)	0.051
HTN (*N* (%))	181 (75.1%)	52 (83.87%)	0.144
Visual impairment (*N* (%))	61 (25.31%)	25 (40.32%)	0.019
Hearing impairment (*N* (%))	23 (9.54%)	12 (19.35%)	0.031
Hyperparathyroidism (*N* (%))	114 (47.3%)	27 (43.55%)	0.597
Intradialytic hypotension (*N* (%))	90 (37.34%)	26 (41.94%)	0.507
Pain (*N* (%))	49 (20.33%)	21 (33.87%)	0.024
Using of walking aids (*N* (%))	25 (10.37%)	16 (25.81%)	0.002
Anxiety, score (IQR)	3 [1;6]	4 [3;6]	0.007
Depression, score (IQR)	4 [2;6]	6 [4;8.75]	<0.001
Weakness, score (IQR)	1 [0;2]	1 [1;2.75]	0.002
SARCF, score (*N* (%))			<0.001
0–4	229 (95.02%)	48 (77.42%)	
5–10	12 (4.98%)	14 (22.58%)	
SPPB, score (*N* (%))			<0.001
0–6	23 (9.54%)	20 (32.26%)	
7–9	74 (30.71%)	19 (30.65%)	
10–12	144 (59.75%)	23 (37.10%)	

BMI: body mass index; DM: diabetes mellitus; HTN: hypertension; SARC-F: Sarcopenia Screening Questionnaire; SPPB: Short Physical Performance Battery Scale.

### Logistic regression

Univariate logistic regression was performed with fall events as the outcome variables, and the results are shown in Table 2. Univariate analysis of age, hearing impairment, using of walking aids, anxiety, depression, weakness, and SPPB showed a significant association with the falls.

**Table 2.** Results of the univariable logistic regression analysis of fall.

Variable	Estimate	Statistic	OR (95% CI)	*p* Value
Age, years	0.04	2.65	1.04 (1.01–1.07)	0.008
Gender				
Male	–	–	1.00 (ref)	–
Female	0.17	0.41	1.18 (0.53–2.67)	0.683
Duration of dialysis, months	0.01	1.18	1.01 (1.00–1.01)	0.238
Educational level				
Primary school	–	–	1.00 (ref)	–
Junior high school	0.74	0.99	2.10 (0.49–9.03)	0.321
Senior high school	−0.18	−0.22	0.83 (0.17–4.09)	0.822
College or higher	−0.09	−0.13	0.91 (0.22–3.70)	0.898
BMI, kg/m^2^	0.01	0.10	1.01 (0.90–1.13)	0.917
DM	0.59	1.41	1.80 (0.80–4.06)	0.159
HTN	0.32	0.64	1.38 (0.52–3.69)	0.522
Visual impairment	0.80	1.93	2.23 (0.99–5.03)	0.053
Hearing impairment	1.42	2.77	4.15 (1.52–11.36)	0.006
Hyperparathyroidism	0.44	1.09	1.55 (0.71–3.40)	0.275
Intradialytic hypotension	0.34	0.83	1.40 (0.63–3.14)	0.408
Pain	0.56	1.25	1.74 (0.73–4.17)	0.212
Using of walking aids	1.20	2.18	3.33 (1.13–9.80)	0.029
Anxiety, score	0.22	2.97	1.24 (1.08–1.43)	0.003
Depression, score	0.32	4.24	1.37 (1.19–1.59)	<0.001
Weakness, score	0.53	2.40	1.70 (1.10–2.63)	0.016
SARC-F, score				
[0–4]	–	–	1.00 (ref)	–
[4–10]	1.15	1.64	3.17 (0.80–12.59)	0.101
SPPB, score				
[9–12]	–	–	1.00 (ref)	–
[6–9]	0.78	1.68	2.18 (0.88–5.42)	0.093
[0–6]	2.00	3.42	7.36(2.34–23.13)	<0.001

BMI: body mass index; DM: diabetes mellitus; HTN: hypertension; SARC-F: Sarcopenia Screening Questionnaire; SPPB: Short Physical Performance Battery Scale.

Variables with *p* < 0.20 in the univariate regression were included in the model for multivariable regression. Full model of multivariable logistic regression is shown in Table 3.

**Table 3.** Results of the full model of multivariable logistic regression.

Variable	Estimate	Statistic	OR (95% CI)	*p* Value
Age, years	0.01	0.55	1.01 (1.06, 0.97)	0.584
DM	0.31	0.59	1.37 (3.88, 0.48)	0.556
Visual impairment	0.27	0.47	1.32 (4.11, 0.42)	0.636
Hearing impairment	0.90	1.35	2.46 (9.06, 0.67)	0.177
Using of walking aids	–0.20	–0.23	0.82 (4.51, 0.15)	0.815
Anxiety, score	–0.13	–0.98	0.88 (1.14, 0.68)	0.325
Depression, score	0.37	2.90	1.45 (1.86, 1.13)	0.004
Weakness, score	–0.09	–0.30	0.91 (1.67, 0.50)	0.762
SARC-F, score				
[0–4]	–	–	1.00 (ref)	–
[4–10]	–1.36	–1.12	0.26 (2.77, 0.02)	0.262
SPPB, score				
[9–12]	–	–	1.00 (ref)	–
[6–9]	–0.19	–0.29	0.83 (3.00, 0.23)	0.773
[0–6]	1.72	1.54	5.56 (49.15, 0.63)	<0.123

DM: diabetes mellitus; SARC-F: Sarcopenia Screening Questionnaire; SPPB: Short Physical Performance Battery Scale.

According to the AIC principle, the lower AIC means the better model (Supplementary File 2). The optimal model was established finally (Table 4). Factors in the optimal model that indicated an increased risk of falls included depression (odds ratio (OR): 1.28, 95% confidence interval (CI): 1.09–1.49, *p* = 0.002) and SPPB <6 (OR: 3.69, 95% CI: 1.04–13.14, *p* = 0.043).

**Table 4.** Results of AIC optimized multivariable logistic regression.

Variable	Estimate	Statistic	OR (95% CI)	*p* Value
Hearing impairment	0.96	1.58	2.62 (0.79–8.66)	0.113
Depression, score	0.25	3.07	1.28 (1.09–1.49)	0.002
SPPB, score				
[9–12]	–	–	1.00 (ref)	–
[6–9]	0.15	0.29	1.17 (0.40–3.37)	0.776
[0–6]	1.31	2.02	3.69 (1.04–13.14)	0.043

SPPB: Short Physical Performance Battery Scale.

### Nomogram construction and validation

**Figure 1.**
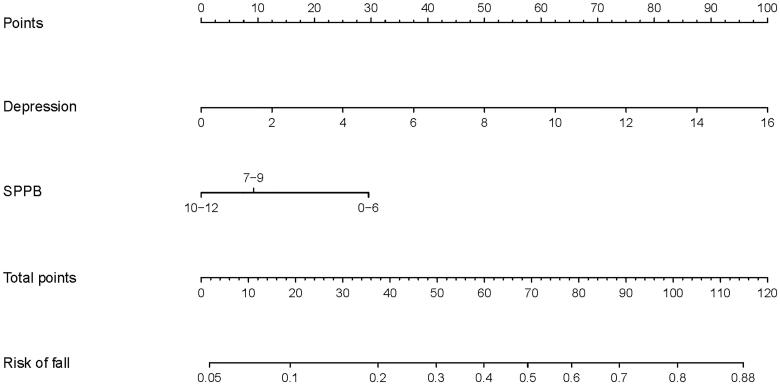
Nomogram of the risk of falls.

Factors that were significant in multivariable regression were used to develop the nomogram to evaluate the risk of falls in MHD patients (Figure 1).

**Figure 2.**
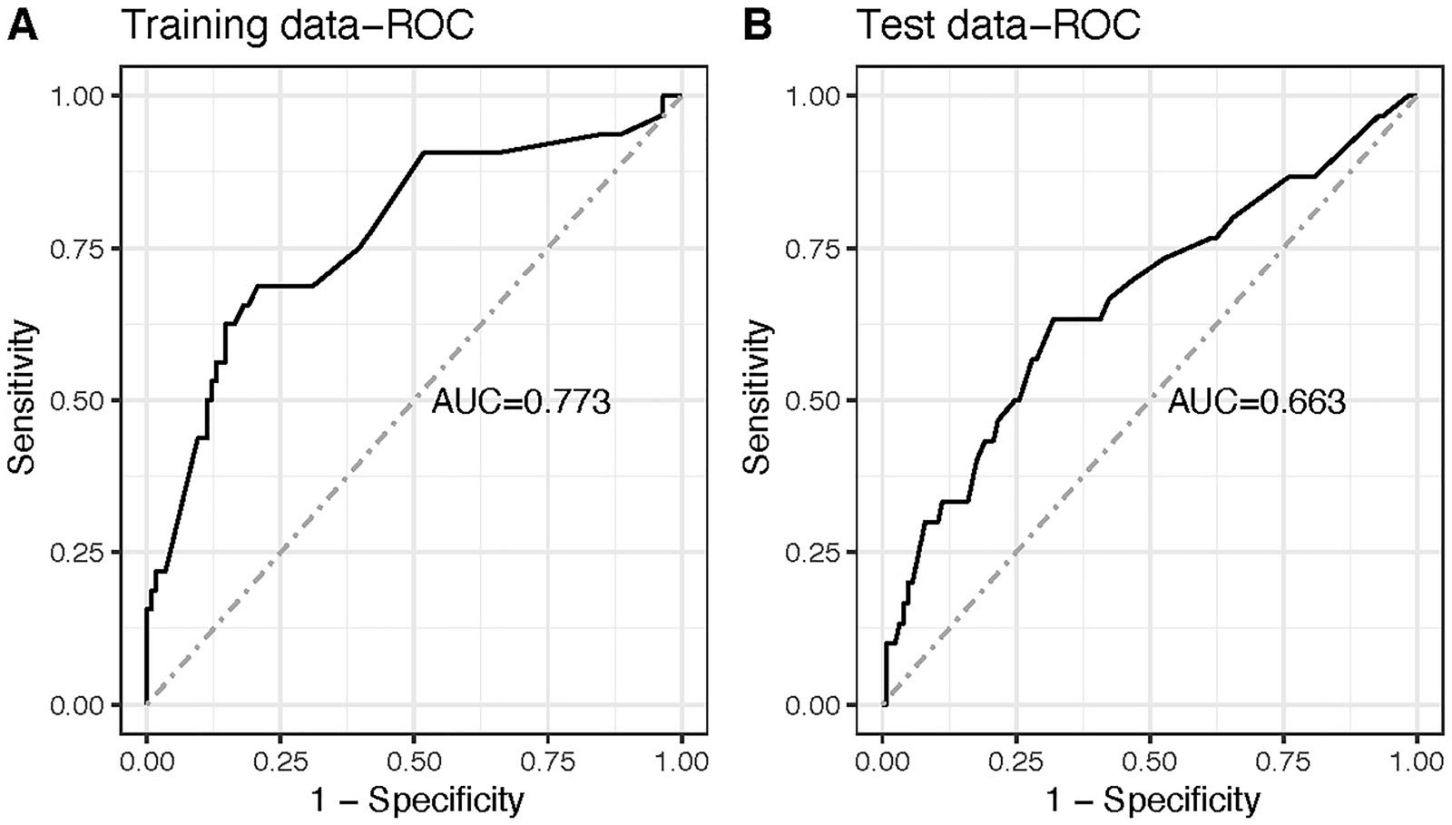
ROC curves of nomogram. Parameters that were analyzed in this regression were depression, and SPPB.

**Figure 3.**
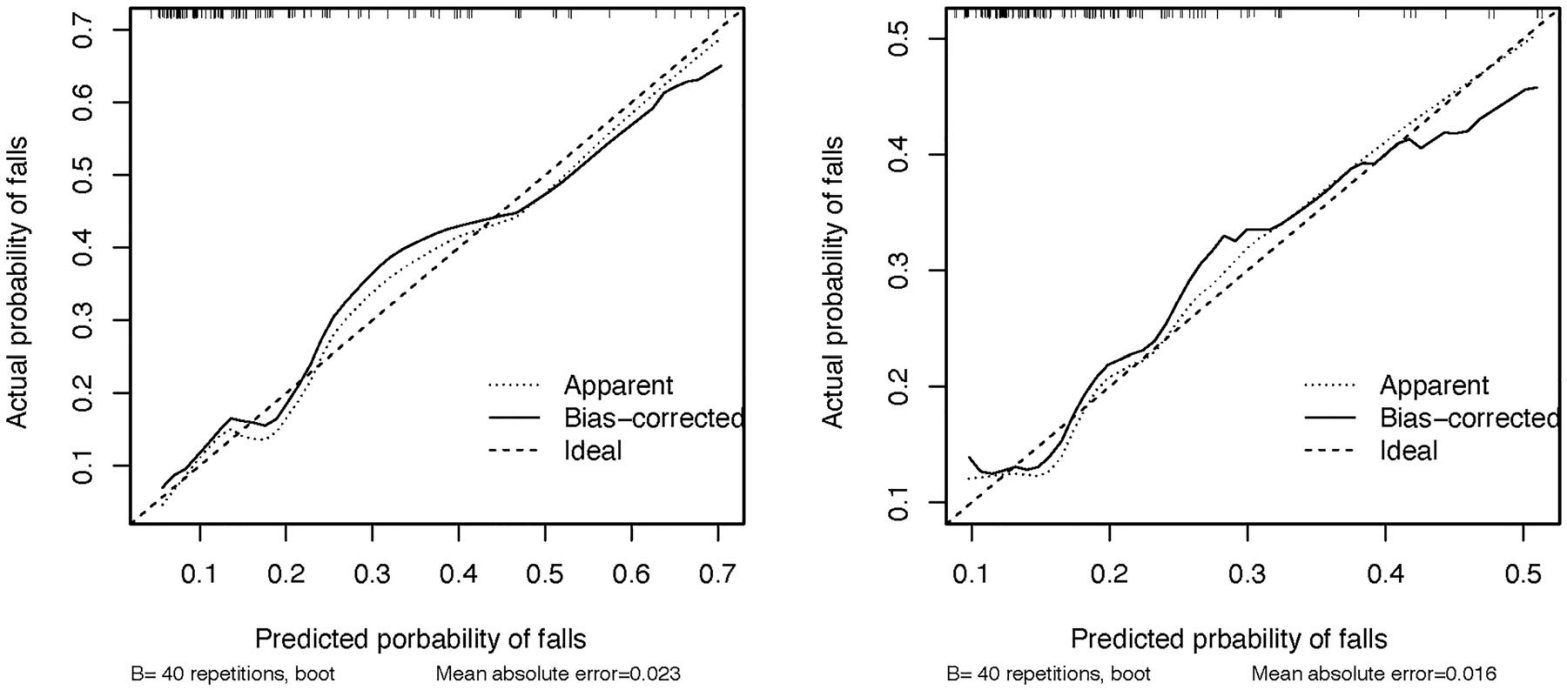
Calibration curves of nomogram. Parameters that were analyzed in this regression were depression, and SPPB.

To determine the accuracy of the models, we plotted the ROC curve of the nomogram in training set and test set (Figure 2). The results suggested that the AUC of the model in the training data was 0.773, with 86.2% sensitivity and 59.4% specificity. AUC of the model in the test data was 0.663, with 76.0% sensitivity and 56.7% specificity. In addition, calibration curves were drawn that showed good agreement between the observed and predicted probabilities in both training and test cohorts (Figure 3).

In order to further verify the effectiveness of nomogram, power analysis was conducted on the model of training group and test group (Table 5). The power of this model of training group and test group was 1.00 and 0.93, respectively.

**Table 5.** Power analysis of nomogram.

	*R* ^2^	Sample size	*f*2	*α*	Power
Training group	0.24	212	0.32	0.05	1.00
Test group	0.12	91	0.13	0.05	0.93

*R*^2^: *R*-square value for logistic model; *f*2: effect size, *f*2 = *R*^2^/(1 – *R*^2^).

### Sensitivity analysis

**Figure 4.**
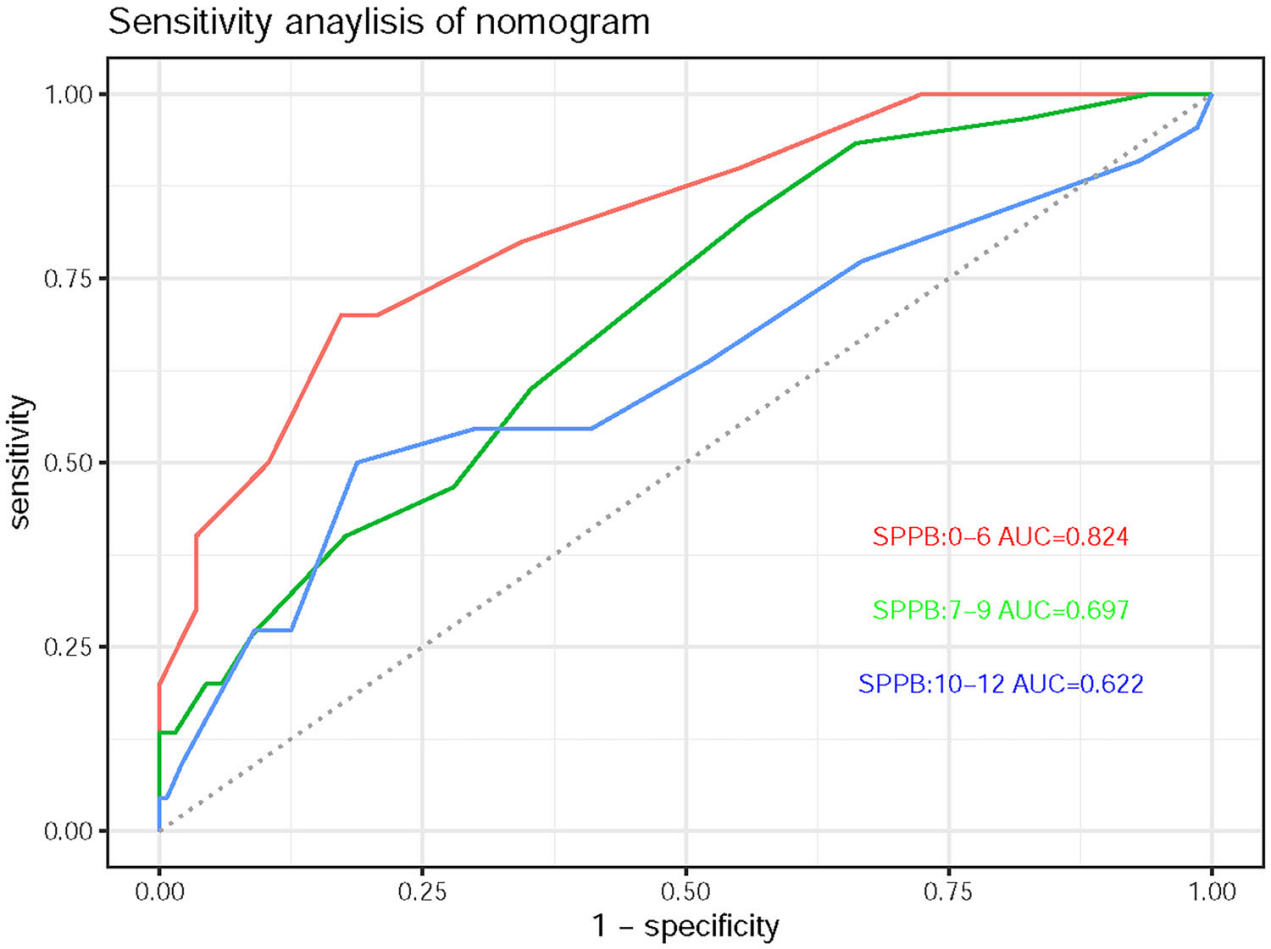
Sensitivity analysis of nomogram.

In order to further verify the robustness of nomogram, we conducted a sensitivity analysis. According to the SPPB score (0–6, 7–9, and 10–12), the subjects were divided into three groups to explore the impact of depression score on the risk of fall (Figure 4). Among the three groups of people with SPPB scores of 0–6, 7–9, and 10–12, the AUCs of nomogram were 0.824, 0.697, and 0.622, respectively.

## Discussion

Long-term dialysis in MHD patients decreases physiological reserves, induces loss of nutrients from the body, and confers a higher risk of falls [20]. Falls not only induce direct harm to patients and prolong the hospitalization duration but also affect the mental health of patients, increase medical costs, and reduce the quality of life [21]. This study investigated the occurrence of falls of 303 patients with MHD, analyzed the risk factors related to falls, and obtained the nomogram to evaluate the risk of falls in MHD patients. We found that depression and SPPB were two important factors affecting the risk of falls in MHD patients.

Many studies have discussed sarcopenia, weakness, etc., in MHD patients [10]. But few studies translated their findings into a clinically model to evaluate the risk of falls in MHD patients. One study established a model of SPPB and Dialysis Fall Risk Index (DFRI) to predict falls in patients with MHD, with an AUC of 0.647 and 0.692, respectively [22]. However, our study further improved the ability to evaluate fall events in MHD patients on the basis of previous models (AUC = 0.773).

This study showed that depressed MHD patients are at high risks of fall. A study showed that the risk of depression in patients with falls increased by 36% compared with those without falls [23]. A meta-analysis also confirmed that there was a correlation between the times of falls and depressive symptoms [24]. Due to the irreversible course of MHD and the long-term treatment process, patients often experience negative emotions. Depression is a common complication of MHD [25]. The prevalence of depression in dialysis patients is nearly five times than that in the general adult population [26]. In other study, the average depression score of MHD patients was 5 [27]. In this study, the median depression score of MHD patients with/without falls was 4 and 6, respectively. According to the nomogram, MHD patients with depression score of 4–6 are at about 14%, 17%, and 21% risk of falls, respectively. If MHD patients with depression score of more than 10, he may be at more than 42% risk of falls. Therefore, in the clinical setting, medical staff should focus on patients with depressive symptoms, and undertake targeted nursing interventions to improve depression and reduce the risk of falls in MHD patients.

The SPPB is a commonly used physical function measurement tool that, in clinical practice, can easily and reliably enable the assessment of balance ability, coordination function, range of motion at joints, reflex control, and so on [28]. SPPB is one of the common tools to assess the risk of falls, but the correlation between SPPB and falls is not the same in different populations. Among the elderly population in the community, the risk of falls with SPPB 4–6 is 1.53 times higher than that with 10–12 [29]. In this study, when only SPPB is considered, the fall risk of patients with 0–6 is 5.56 times that of patients with 10–12. According to the nomogram, MHD patients with depression score of 0–6 are at about 16% risk of falls. Therefore, medical staff should regularly use the SPPB scale to measure the physical function of MHD patients, so as to understand the patient’s exercise ability, reaction ability and muscle condition. They should provide guidance to patients with SPPB score of 7–9 in time to help restore body function; give special guidance and care to patients with SPPB ≤ 6 to reduce the occurrence of falls.

In the sensitivity analysis of nomogram, among the three groups of people with SPPB scores of 0–6, 7–9, and 10–12, the AUCs of nomogram were 0.824, 0.697, and 0.622. This suggests that nomogram has better evaluation ability among MHD patients with SPPB ≤ 6. Actually, among MHD patients with SPPB ≤ 6, the risk of falls may increase from 16% to more than 88% with depression. This suggests that we should make full use of this nomogram tool, to evaluate the emotional state of MHD patients with SPPB ≤ 6.

### Strength and limitation

As we know, this study may be the first one to use two easily available variables to evaluate the risk of falls in MHD patients in China. Our study further improved the ability to evaluate fall events in MHD patients on the basis of previous models (AUC = 0.773 vs. AUC = 0.692).

Some factors are of interest in relation to falls in MHD patients, such as albumin, hemoglobin, medication use (like drugs affecting the central nervous system, antihypertensives, and cardiovascular medications), etc. The relationship between these factors and falls has been inconsistent across studies [30–32]. However, unfortunately we were not able to collect this information in our study. We look forward to exploring these factors further in future studies.

What is more, the AUC of nomogram in this study is 0.773, which is better than previous studies, but still has room for further improvement. We look forward to large sample, prospective cohort studies to further verify our result.

## Supplementary Material

Supplemental Material
