# Supplementary material for: Novel risk-factor analysis and risk-evaluation model of falls in patients receiving maintenance hemodialysis
Source: Ren Fail. 2023 Mar 1;45(1):2182608. doi: 10.1080/0886022X.2023.2182608 (PMC9980417; doi:10.1080/0886022X.2023.2182608)
Supplement: Supplemental Material [file IRNF_A_2182608_SM6055.pdf]

Study ID ______________________ Date ____________________ Tester Initials ____________________

SHORT PHYSICAL PERFORMANCE BATTERY PROTOCOL AND SCORE SHEET

All of the tests should be performed in the same order as they are presented in this protocol. Instructions to the participants are shown in bold italic and should be given exactly as they are written in this script.

1. BALANCE TESTS

The participant must be able to stand unassisted without the use of a cane or walker. You may help the participant to get up.

> ***Now let's begin the evaluation. I would now like you to try to move your body in different movements. I will first describe and show each movement to you. Then I'd like you to try to do it. If you cannot do a particular movement, or if you feel it would be unsafe to try to do it, tell me and we'll move on to the next one. Let me emphasize that I do not want you to try to do any exercise that you feel might be unsafe.***
>
> ***Do you have any questions before we begin?***

A. Side-by-Side Stand

1. ***Now I will show you the first movement***.
2. (Demonstrate) ***I want you to try to stand with your feet together, side-by-side, for about 10 seconds.***
3. ***You may use your arms, bend your knees, or move your body to maintain your balance, but try not to move your feet. Try to hold this position until I tell you to stop.***
4. Stand next to the participant to help him/her into the side-by-side position.
5. Supply just enough support to the participant's arm to prevent loss of balance.
6. When the participant has his/her feet together, ask ***"Are you ready?"***
7. Then let go and begin timing as you say, ***"Ready, begin."***
8. Stop the stopwatch and say ***"Stop"*** after 10 seconds or when the participant steps out of position or grabs your arm.
9. If participant is unable to hold the position for 10 seconds, record result and go to the gait speed test.

B. Semi-Tandem Stand

1. ***Now I will show you the second movement***.

Information Classification: General

2. (Demonstrate) ***Now I want you to try to stand with the side of the heel of one foot touching the big toe of the other foot for about 10 seconds. You may put either foot in front, whichever is more comfortable for you.***

3. ***You may use your arms, bend your knees, or move your body to maintain your balance, but try not to move your feet. Try to hold this position until I tell you to stop.***

4. Stand next to the participant to help him/her into the semi-tandem position

5. Supply just enough support to the participant's arm to prevent loss of balance.

6. When the participant has his/her feet together, ask ***"Are you ready?"***

7. Then let go and begin timing as you say ***"Ready, begin."***

8. Stop the stopwatch and say ***"Stop"*** after 10 seconds or when the participant steps out of position or grabs your arm.

9. If participant is unable to hold the position for 10 seconds, record result and go to the gait speed test.

C. Tandem Stand

1. ***Now I will show you the third movement***.

2. (Demonstrate) ***Now I want you to try to stand with the heel of one foot in front of and touching the toes of the other foot for about 10 seconds. You may put either foot in front, whichever is more comfortable for you.***

3. ***You may use your arms, bend your knees, or move your body to maintain your balance, but try not to move your feet. Try to hold this position until I tell you to stop.***

4. Stand next to the participant to help him/her into the tandem position.

5. Supply just enough support to the participant's arm to prevent loss of balance.

6. When the participant has his/her feet together, ask ***"Are you ready?"***

7. Then let go and begin timing as you say, ***"Ready, begin."***

8. Stop the stopwatch and say ***"Stop"*** after 10 seconds or when the participant steps out of position or grabs your arm.

SCORING:

Study ID ______________________ Date ___________________ Tester Initials ____________________

If participant did not attempt test or failed, circle why:

Tried but unable	1
Participant could not hold position unassisted	2
Not attempted, you felt unsafe	3
Not attempted, participant felt unsafe	4
Participant unable to understand instructions	5
Other (specify) ___________________________	6
Participant refused	7

A. Side-by-side-stand

Held for 10 sec ❒ 1 point

Not held for 10 sec ❒ 0 points Not attempted

❒ 0 points

If 0 points, end Balance Tests

Number of seconds held if less than 10 sec: __.__ __sec

B. Semi-Tandem Stand

Held for 10 sec ❒ 1 point

Not held for 10 sec ❒ 0 points

Not attempted ❒ 0 points *(circle reason above)*

If 0 points, end Balance Tests

Number of seconds held if less than 10 sec: __.__ sec

C. Tandem Stand

Held for 10 sec ❒ 2 points

Held for 3 to 9.99 sec ❒ 1 point

Held for < than 3 sec ❒ 0 points

Not attempted ❒ 0 points (circle reason above)

Number of seconds held if less than 10 sec: __.__ sec

D. Total Balance Tests score __________ **(sum points)**

Comments:

____

____

____

____

____

Information Classification: General

Study ID ________________________ Date ____________________ Tester Initials ______________________

2. GAIT SPEED TEST

Now I am going to observe how you normally walk. If you use a cane or other walking aid and you feel you need it to walk a short distance, then you may use it.

A. First Gait Speed Test

1. ***This is our walking course. I want you to walk to the other end of the course at your usual speed, just as if you were walking down the street to go to the store.***
2. Demonstrate the walk for the participant.
3. ***Walk all the way past the other end of the tape before you stop. I will walk with you. Do you feel this would be safe?***
4. Have the participant stand with both feet touching the starting line.
5. ***When I want you to start, I will say: "Ready, begin."*** When the participant acknowledges this instruction say: ***"Ready, begin."***
6. Press the start/stop button to start the stopwatch as the participant begins walking.
7. Walk behind and to the side of the participant.
8. Stop timing when one of the participant's feet is completely across the end line.

B. Second Gait Speed Test

1. ***Now I want you to repeat the walk. Remember to walk at your usual pace, and go all the way past the other end of the course.***
2. Have the participant stand with both feet touching the starting line.
3. ***When I want you to start, I will say: "Ready, begin."*** When the participant acknowledges this instruction say: ***"Ready, begin."***
4. Press the start/stop button to start the stopwatch as the participant begins walking.
5. Walk behind and to the side of the participant.
6. Stop timing when one of the participant's feet is completely across the end line.

GAIT SPEED TEST SCORING:

Information Classification: General

Study ID ________________________ Date ____________________ Tester Initials ______________________

Length of walk test course: Four meters ❒ Three meters ❒

A. Time for First Gait Speed Test (sec)

1. Time for 3 or 4 meters __ __.__ __ sec
2. If participant did not attempt test or failed, circle why:

   Tried but unable 1

   Participant could not walk unassisted 2

   Not attempted, you felt unsafe 3

   Not attempted, participant felt unsafe 4

   Participant unable to understand instructions5

   Other (Specify) ________________________ 6

   Participant refused 7

   Complete score sheet and go to chair stand test

3. Aids for first walk……………None ❒ Cane ❒ Other ❒ Comments:

____

____

____

B. Time for Second Gait Speed Test (sec)

1. Time for 3 or 4 meters __ __.__ __ sec
2. If participant did not attempt test or failed, circle why:

   Tried but unable 1

   Participant could not walk unassisted 2

   Not attempted, you felt unsafe 3

   Not attempted, participant felt unsafe 4

   Participant unable to understand instructions5

   Other (Specify) 6

   Participant refused 7

3. Aids for second walk……….. None ❒ Cane ❒ Other ❒

What is the time for the faster of the two walks?

Record the shorter of the two times __ __.__ __ sec

[If only 1 walk done, record that time] __ __.__ __ sec

If the participant was unable to do the walk: ❒ **0 points**

For 4-Meter Walk:

If time is more than 8.70 sec: ❒ **1 point**

If time is 6.21 to 8.70 sec: ❒ **2 points**

For 3-Meter Walk:

If time is more than 6.52 sec: ❒ **1 point**

If time is 4.66 to 6.52 sec: ❒ **2 points**

Information Classification: General

If time is 4.82 to 6.20 sec:	❒ **3 points**	If time is 3.62 to 4.65 sec:	❒ **3 points**
If time is less than 4.82 sec:	❒ **4 points**	If time is less than 3.62 sec:	❒ **4 points**

3. CHAIR STAND TEST Single Chair Stand

1. ***Let's do the last movement test. Do you think it would be safe for you to try to stand up from a chair without using your arms?***

2. ***The next test measures the strength in your legs.***

3. (Demonstrate and explain the procedure.) ***First, fold your arms across your chest and sit so that your feet are on the floor; then stand up keeping your arms folded across your chest.***

4. ***Please stand up keeping your arms folded across your chest.*** (Record result).

5. If participant cannot rise without using arms, say ***"Okay, try to stand up using your arms."*** This is the end of their test. Record result and go to the scoring page.

Repeated Chair Stands

1. ***Do you think it would be safe for you to try to stand up from a chair five times without using your arms?***

2. (Demonstrate and explain the procedure): ***Please stand up straight as QUICKLY as you can five times, without stopping in between. After standing up each time, sit down and then stand up again. Keep your arms folded across your chest. I'll be timing you with a stopwatch.***

3. When the participant is properly seated, say: ***"Ready? Stand"*** and begin timing.

4. Count out loud as the participant arises each time, up to five times.

5. Stop if participant becomes tired or short of breath during repeated chair stands.

6. Stop the stopwatch when he/she has straightened up completely for the fifth time.

7. Also stop:
   - If participant uses his/her arms
   - After 1 minute, if participant has not completed rises
   - At your discretion, if concerned for participant's safety

8. If the participant stops and appears to be fatigued before completing the five stands, confirm this by asking ***"Can you continue?"***

9. If participant says "Yes," continue timing. If participant says "No," stop and reset the stopwatch.

Information Classification: General

Study ID ____________ Date ____________ Tester Initials ____________

SCORING Single Chair Stand Test

		YES	NO
A.	Safe to stand without help	❒	❒

B. Results:

Participant stood without using arms	❒	→ Go to Repeated Chair Stand Test
Participant used arms to stand	❒	→ End test; score as 0 points
Test not completed	❒	→ End test; score as 0 points

C. If participant did not attempt test or failed, circle why:

Tried but unable	1
Participant could not stand unassisted	2
Not attempted, you felt unsafe	3
Not attempted, participant felt unsafe	4
Participant unable to understand instructions	5
Other (Specify) ____________	6
Participant refused	7

Repeated Chair Stand Test

		YES	NO
A.	Safe to stand five times	❒	❒

B. If five stands done successfully, record time in seconds.

Time to complete five stands __ __.__ __ sec

C. If participant did not attempt test or failed, circle why:

Tried but unable	1
Participant could not stand unassisted	2
Not attempted, you felt unsafe	3
Not attempted, participant felt unsafe	4
Participant unable to understand instructions	5

Information Classification: General

Other (Specify)	6
Participant refused	7

Scoring the Repeated Chair Test

Participant unable to complete 5 chair stands or completes stands in >60 sec: ❒ 0 points

If chair stand time is 16.70 sec or more: ❒ 1 points If chair stand time is 13.70 to 16.69 sec: ❒ 2 points

If chair stand time is 11.20 to 13.69 sec: ❒ 3 points

If chair stand time is 11.19 sec or less: ❒ 4 points

Study ID Date Tester Initials

Scoring for Complete Short Physical Performance Battery

Test Scores Total Balance Test score _____ points Gait Speed Test score _____ points Chair Stand Test score _____ points

Total Score _____ points (sum of points above)

Information Classification: General
